# Direct Arylation Strategies in the Synthesis of π-Extended Monomers for Organic Polymeric Solar Cells

**DOI:** 10.3390/molecules22010021

**Published:** 2016-12-26

**Authors:** Andrea Nitti, Riccardo Po, Gabriele Bianchi, Dario Pasini

**Affiliations:** 1 Department of Chemistry, University of Pavia, Viale Taramelli, 12, 27100 Pavia, Italy; andrea.nitti01@universitadipavia.it; 2 Research Center for Renewable Energies & Environment, Eni spa, Via Giacomo Fauser 4, 28100 Novara, Italy; riccardo.po@eni.com (R.P.); Gabriele.Bianchi1@eni.com (G.B.); 3 INSTM Research Unit, University of Pavia, Viale Taramelli, 12, 27100 Pavia, Italy

**Keywords:** π-conjugated monomers, π-conjugated polymers, Direct Arylation, organic photovoltaics, palladium catalysts

## Abstract

π-conjugated macromolecules for organic polymeric solar cells can be rationally engineered at the molecular level in order to tune the optical, electrochemical and solid-state morphology characteristics, and thus to address requirements for the efficient solid state device implementation. The synthetic accessibility of monomers and polymers required for the device is getting increasing attention. Direct arylation reactions for the production of the π-extended scaffolds are gaining importance, bearing clear advantages over traditional carbon-carbon forming methodologies. Although their use in the final polymerization step is already established, there is a need for improving synthetic accessibility to implement them also in the monomer synthesis. In this review, we discuss recent examples highlighting this useful strategy.

## 1. Introduction

Since the discovery of highly conducting polyacetylene by Shirakawa et al. [1] in 1977, conjugated π-extended organic systems based on aromatic and heteroaromatic compounds have been receiving increasing attention as promising materials for the development of the new generation of organic electronic and optoelectronic materials [2]. The huge potential offered by organic synthesis for the flexible construction of conjugated π-extended compounds, coupled with the unraveling of molecular design concepts, have led to a large number of all-organic electronic and optoelectronic devices such as field-effect transistors (FET) [3], light-emitting diodes (LED) [4], photovoltaics [5] and optical communication devices [6].

Organic Photovoltaic devices (OPV) with bulk-heterojunction architecture (BHJ) have attracted global attention for their unique features, which include tunable photoelectronic properties of the active layer, low cost, light weight, flexibility, and large area production with roll-to-roll printing techniques [7]. Recent progress in this area afforded in 2016 a record high power-conversion efficiency (PCE) of 11.5% [8,9]. From an industrial point of view, the ultimate legitimation and large scale commercialization OPV-BHJ devices will need PCE over the critical threshold of 10%, printability on a large area of the mixture donor polymers/acceptor fullerenes, as well as efficient and scalable syntheses of the donor polymers [10].

A large number of conjugated polymers alternating π-electron donor and π-electron acceptor units (D–A polymers) have been proposed in the literature [11], most of which were obtained via the well-known Suzuki and Stille cross-coupling polymerizations, and make use of organoboron and tin derivatives, respectively [12,13]. In both cases, as the polymerization mechanism is through polycondensation, thorough purification of the monomers is mandatory in order to ensure high purity, which in turn is essential for assuring stoichiometric equivalence of the reactive functional groups to be able to achieve high conversion and high degrees of polymerizations. There are numerous benefits in the use of these polymerization procedures: standardized protocols, tolerance of many functional groups, commercial availability of common organoboron and tin derivatives. Their popularity is therefore still high in lab-scale productions. However, in the perspective of industrialization and large-scale production, the use of these methodologies may be limited by the use of biphasic solvents or the need to protect sensitive groups in case of the Suzuki procedures, involving organoborons, and by the production of toxic byproducts in the case of the Stille polymerization procedures.

**Figure 1:**
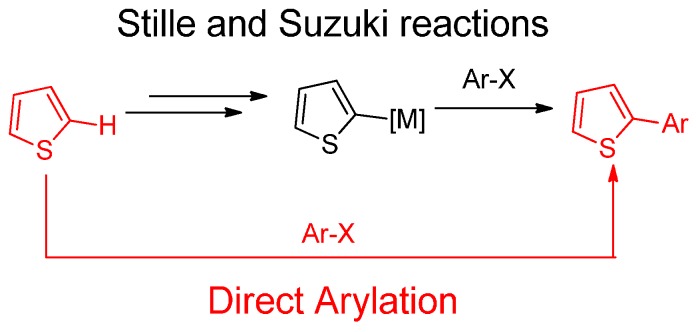
General Scheme for direct arylation reactions.

In recent years, direct hetero arylation polycondensation (DHAP) of non-preactivated arenes with aryl halides or pseudohalides has attracted much attention and worldwide interest as an alternative to Stille and Suzuki reactions [14]. From industrial point of view, DHAP protocols bear indisputable advantages: synthetic simplicity, requiring cheap, readily available catalysts, and atom economy, since the preactivation of the donor component (Figure 1) using (hetero)aryl organometallic intermediates is not required. These indisputable advantages have prompted the exploitation of DHA reactions as protocols for polymerizations, and several successful examples have already been reported in the literature.

A recent, thorough analysis of the published strategies for the synthesis of OPV-BHJ conjugated polymers has introduced the “synthetic accessibility” parameter, useful for their direct comparison, in the light of their potential scalability and commercialization. This parameter clearly suggests that the synthesis of the monomers themselves is a key element for the scalability of the resultant donor polymer [15]. “Synthetic accessibility” takes into consideration: (a) the number of synthetic steps; (b) the cost of the materials; (c) yields; (d) the number of steps of purification that require column chromatography; (e) the inherent safety of reagents. The use of DHA reactions for the construction of the monomer themselves could lead to important benefits on all parameters that define “synthetic accessibility”.

In this review, we will highlight recent examples of the use of DHA reactions for the construction of π-extended monomers, in order to establish a link between the current use of this relatively recent synthetic methodology, and the development of scalable monomers for OPV-BHJ low-cost donor polymers. We refer the reader to section 5 for a list of abbreviations used throughout the text. We will not deal with the synthesis via DHA of π-extended compounds used for the fabrication of small molecule solar cells or dye-sensitized solar cells (DSSCs). We refer the reader to a review on the subject [16], and related recent examples [17,18,19].

## 2. Discussion

**Scheme 1:**
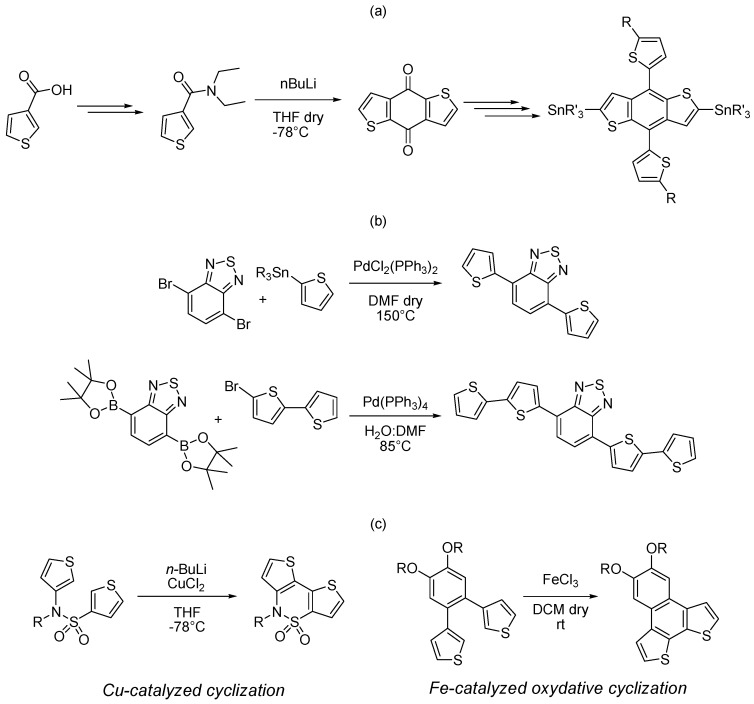
Typically crucial step in the synthesis of monomers: (**a**) α-lithiation for the synthesis of DTBDT (dithienobenzodithiophene); (**b**) Stille and Suzuki protocols; (**c**) transition-metal catalyzed methodologies. DMF: *N*,*N*-dimethylformamide; NDT: naphto[2,1-*b*:3,4-*b*′]dithiophene. DCM: dichloromethane. THF: tetrahydrofuran.

The crucial step in the synthesis of the π-extended monomers is the formation of the carbon framework, which is generally achieved utilizing reactions carried out between an intermediate organometallic species and its electrophilic partners. The synthesis of the organometallic species generally leads to an increment of the number of steps. A typical example of this strategy is the α-lithiation of *N*,*N*-diethyl-3-carboxythiophene for the reaction with itself to give the dithienobenzodithiophene (DTBDT) framework [20] (Scheme 1a). Cross-coupling methodologies, such as Stille and Suzuki protocols, may use commercial available organotin and organoboron species for the achievement of target molecules (Scheme 1b) [21,22]. The formation of inorganic or organometallic byproducts with intrinsic toxicity, especially for the trialkyltin derivatives used in the Stille protocols, require mandatorily the use of column chromatography as the purification method. Furthermore, similarly to Grignard or organolithium compounds, when the halide-intermediate and organotin or organoboron species are not commercially available, further synthetic steps are required.

Transition-metal catalyzed methodologies that do not make use of organometallic reagents such as Cu-[23] or Fe-catalyzed [24] oxidative couplings have shown potential in the construction of the carbon frameworks of high-efficiency monomers (Scheme 1c).

When compared to the previously mentioned examples, DHA reactions are attracting increasing attention as a Pd-catalyzed cross-coupling methodology since no pre-activation of the starting materials with organometallic reagents is required; they are regioselective, efficient and column chromatography purifications can be avoided. Despite the fact that Stille and Suzuki protocols require a lower loading of the catalyst (2–10 mol % vs. 5–20 mol % in the case of DHA), DHA reactions improve synthetic accessibility of the final monomers reducing the number of synthetic steps, the cost of the materials and avoiding the formation of toxic byproducts.

**Figure 2:**
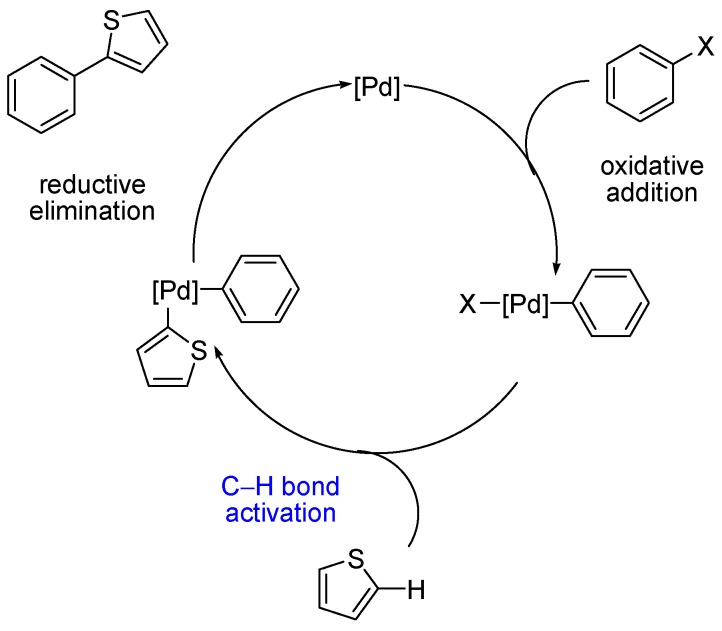
Catalytic cycle for Pd-catalyzed direct hetero arylation (DHA) reaction and possible transition states for C–H bond activation.

Direct arylation has been efficiently applied to the coupling of a number of different arene and heteroarene systems [25]. In particular, the activation of thiophene C–H bonds is the most studied due to importance of this heterocycle for the rational design of π-extended monomers for organic electronics. The catalytic cycle for Pd-catalyzed direct arylation involving thiophene rings is illustrated in Figure 2 and consists of three steps: oxidative addition, C–H bond activation and reductive elimination.

The mechanism by which C–H activation occurs has been widely studied and base-assisted concerted metalation-deprotonation (CMD) is believed to be the pathway followed by thiophene-based compounds because of its small activation energy [26].

We will divide literature approaches into two categories: (1) intermolecular DHA and (2) intramolecular DHA.

### 2.1. Intermolecular DHA

An important class of high performance dyes, 2,1,3-benzothiadiazole (BT) and its derivatives, have in recent years been widely explored for optoelectronic applications because of their unique electrical and optical properties, as well as their exceptional stability. BT derivatives are generally synthesized through Stille or Suzuki coupling reactions using different organometallic reagent and 4,7-dibromo-2,1.3-benzothiadiazole (DBrBT) **2**. It is not thus surprising that much of the literature on DHA reactions is focused on the synthesis of BT derivatives.

**Scheme 2:**
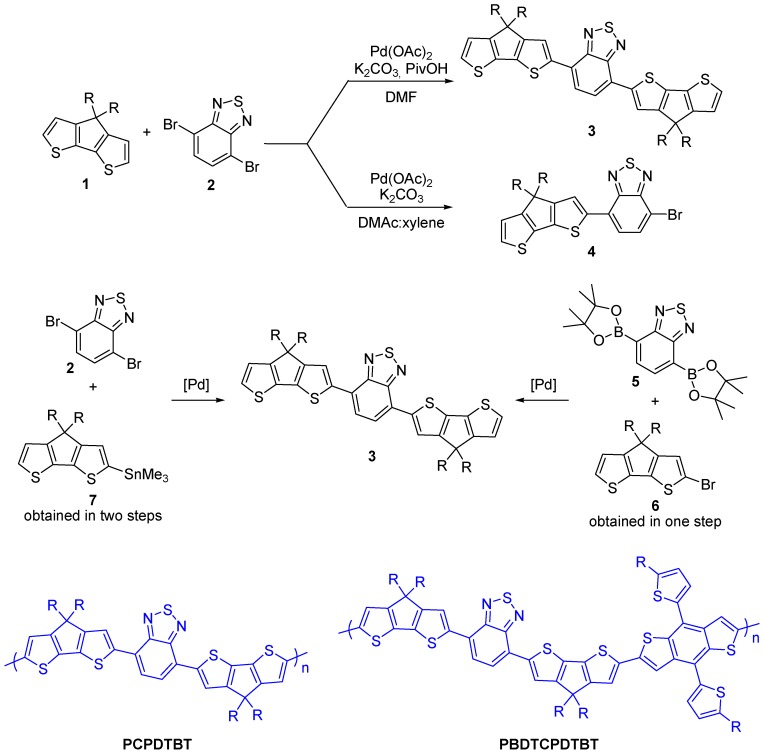
DHA reaction between 4*H*-cyclopenta[1,2-*b*:5,4-*b*′]dithiophene (CPDT) and 2,1,3-benzothiadiazole (BT) compounds and their selected polymers. DMAc: *N*,*N*-dimethylacetamide.

M. Horie et al. [27] reported an interesting DHA reaction between DBrBT **2** (Scheme 2) and alkylated 4H-cyclopenta[1,2-*b*:5,4-*b*′]dithiophene (CPDT) **1** for the construction of a monomeric unit to be incorporated in several polymers with high hole mobility and PCE around 5%–6% such as PCPDTBT or PBDTCPDTBT [28,29].

The synthesis of oligomeric monomer **3**, shown in Scheme 2, was achieved with a 40% yield using Pd(OAc)_2_ as catalyst (25 mol %) in presence of K_2_CO_3_ as base, PivOH as additive and *N*,*N*-dimethylformamide (DMF). The same author reported the synthesis of **3** via Suzuki coupling (60% yield) using Pd(PPh_3_)_4_ as catalyst system (30 mol %), commercial 2,1,3-Benzothiadiazole-4,7-bis(pinacol boronate ester) **5** and mono-brominated CPDT **6**, which had to be prepared via bromination reaction with *N*-bromosuccinimide (NBS) carried out on compound **1**. The synthesis of **3** could be achieved with Stille protocol [30] using 5 mol % of Pd(PPh_3_)_4_ but, in analogy with the Suzuki protocol, the synthesis of starting material **7** was needed. The procedure in Scheme 2 highlights a greater synthetic sustainability of DHA compared to Suzuki and Stille reactions in terms of reduction of synthetic steps, reaction time, *E*-factor (with reduction of wastes) and cost of reactions in spite of a yield decrease.

**Scheme 3:**
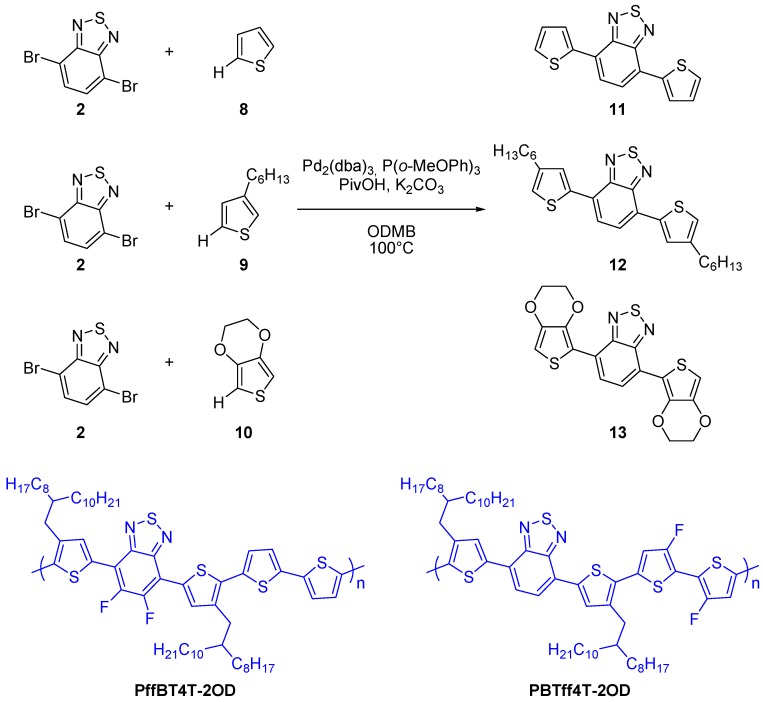
Synthesis of 4,7-di(thiophen-2-yl)-2,1,3-benzothiadiazole (DTBT) and its high-efficiency polymerspoly[(5,6-difluoro-2,1,3-benzothiadiazol-4,7-diyl)-alt-(3,3‴-di(2-octyldodecyl)-2,2′;5′,2″;5″,2‴-quaterthiophen-5,5‴-diyl)] (PffBT4T-2OD) and poly[(2,1,3-benzothiadiazol-4,7-diyl)-alt-(3,3‴-di(2-octyldodecyl)-3′3″-difluoro-2,2′;5′,2″;5″,2‴-quaterthiophen-5,5‴-diyl)] (PDTff4T-2OD). ODMB: *o*-dimethoxybenzene.

Wang et al. [31] reported the synthesis of a series of 4,7-di(thiophen-2-yl)-2,1,3-benzothiadiazole (DTBT) monomers **11** (DTBT), **12** (4,7-bis(4-hexylthiophen-2-yl)-2,1,3-benzothiadiazole, DHTBT) and **13** (4,7-bis(3,4-ethylenedioxythiophene-2-yl)-2,1,3-benzothiadiazole, DEDOTBT) with a Pd_2_(dba)_3_-catalyzed DHA protocol using commercially-available **2**, **8**, **9** and **10** (Scheme 3). DTBTs are one of the most important building blocks in conjugated polymers for high-performance optoelectronic devices; the highest PCE achieved to date in a OPV-BHJ cell (11.5%) was obtained from a polymer (PBTff4T-2OD in Scheme 3) having DTBT unit as the acceptor of the D-A scaffold [8].

The DHA reaction showed lower efficiencies (compounds **11**, **12** and **13** were obtained with yields of 67%, 76% and 43%, respectively) when compared with the efficiencies of Stille and Suzuki protocols (yield 80%–90%) [32,33], due to the inevitable formation of byproducts.

**Figure 3:**
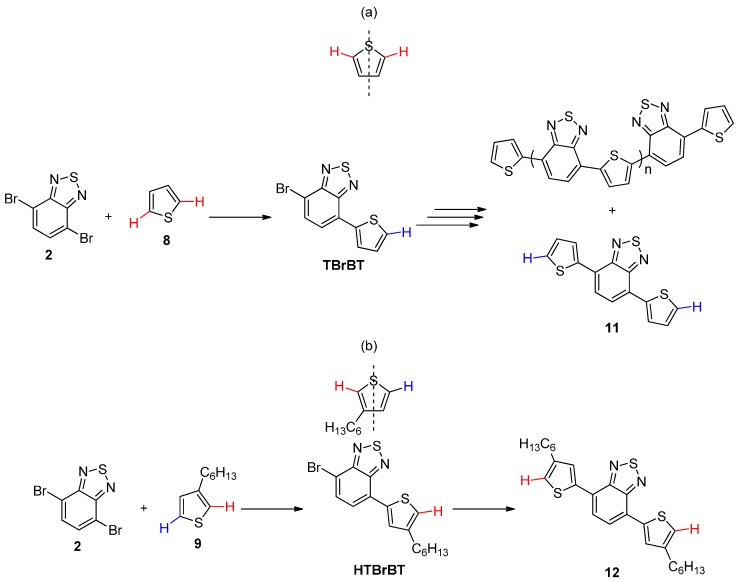
Possible reaction pathways and side reactions in the formation of monomers **11** and **12** through DHA protocols. TBrBT: 4-thiophene-7-bromo-2,1.3-benzothiadiazole; HTBrBT: 4-(3-hexylthiophene)-7-bromo-2,1.3-benzothiadiazole; (**a**) intermolecular DHA for monomer **11**; (**b**) intermolecular DHA for monomer **12**.

The authors reported, even in optimized conditions, the formation of insoluble and partially insoluble purple byproducts (oligomers and polymers) when **2** was reacted with **8** and **10**. The formation of these higher molecular weight byproducts was explained with a greater reaction rate of the product **11** and intermediate TBrBT (4-thiophene-7-bromo-2,1.3-benzothiadiazole, Figure 3a, blue bonds) towards aryl bromide species with respect to the reaction rate involving bare thiophene **8**. Increasing the Brønsted acidity of the remaining α–H on the thiophene ring of **11** and intermediate TBrBT compared to bare thiophene **8** (Figure 3a, red bonds) is likely the cause of the formation of oligomers and polymers. The reaction between **2** with symmetric **10** to form **13** was also affected by formation of a high molecular weight byproducts in analogy to the precedent case, while the reaction of **2** with asymmetric **9** proceeded to form **12** in good yields, with a suppressed formation of byproducts (Figure 3b). The authors ascribe this to the different reactivity of the 2- and 5-position C–H bonds on **9**. In fact, the C5–H bond on **9** (Figure 3b, blue bonds) is more reactive toward DHA reactions than the C2–H bond on **9** and the C5–H bond on HTBrBT intermediate (4-(3-hexylthiophene)-7-bromo-2,1.3-benzothiadiazole, Figure 3b, red bonds).

These examples showed an important limitation of the use of intermolecular DHA protocols when symmetric compounds (such as **1**, **8** and **10**) are used. In such cases, both α–H position on thiophene rings are equally reactive toward DHA reaction, and intermediates with a more reactive α–H than its precursor are formed, with the consequent formation of oligomeric byproducts. When the α–H are unsymmetrically reactive towards the DHA reaction, an intermediate with a less reactive α–H than its precursor may be formed, with improvement in the yield of reaction. In general, the intermolecular DHA reaction works well if the intermediate species have a less reactive α–H than its precursor, otherwise the intermolecular DHA reaction will show a decrement of yield due to the formation of oligomers.

**Scheme 4:**
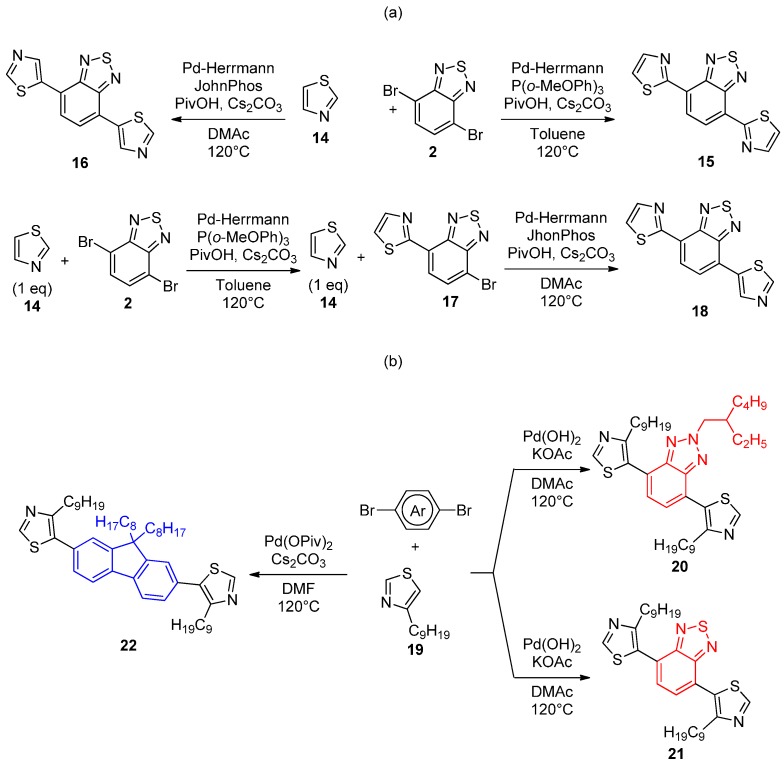
Synthesis of thiazole-based monomers via DHA reaction. BT: 2,1,3-benzothiadiazole, BTZ: 1,2,3-benzotriazole; (**a**) intermolecular DHA between **2** and commercial thiazole; (**b**) intermolecular DHA with electron-poor arene units.

These considerations have further confirmation in the work of Leclerc et al. [34] on intermolecular DHA between **2** and commercial thiazole **14** to give monomers **15** and **16,** respectively, with the thiazole unit linked with its C2 position and C5 position with high regioselectivity (Scheme 4a). The thiazole unit has non-equivalent C2–H and C5–H positions that can be each activated towards DHA using different reaction conditions. The asymmetric reactivity of the two α–H positions can promote the formation, using the conditions for the synthesis of **15** but using only one equivalent of thiazole **14**, of compound **17**, possessing a less reactive α–H than precursor **14** toward DHA reaction conditions and avoiding oligomerization. The authors obtained the highly regioselective C2–C2 product **15** (**15**:**16** ratio 99:1, 79% yield) using an apolar solvent (toluene) and Herrmann’s catalyst with the less bulky added ligand tris(*o*-methoxyphenyl)phosphine, whereas the use a polar solvent (*N*,*N*-dimethylacetamide, DMAc) and Pd-Herrmann catalyst with the bulkier ligand JohnPhos led to highly regioselective C5–C5 products **16** (**15**:**16** ratio 1:99, 75% yield). In both cases the authors did not observe any oligomerization, indicating good regiocontrol in the DHA reaction. The author reported the synthesis of C2–C5 monomer **18** starting from isolated compound **17**.

You et al. [35] obtained monomer **20** (62% yield) and **21** (77% yield), with electron-poor arene units (2,1,3-benzothiadiazole, BT unit and 1,2,3-benzotriazole, BTZ unit), using Pd(OH)_2_ as the catalyst (10 mol %) in DMAc, while for monomer **22** (72% yield), which present an electron-rich F unit, Pd(OPiv)_2_ (10 mol %) was used as the catalyst in DMF (Scheme 4b).

### 2.2. Intramolecular DHA

Intramolecular DHA reactions have been used for the construction of novel monomer frameworks due to their reliability and the possibility of standardization of the process. The protocols developed for these reactions are generally conducted without the use of expensive phosphines and using Pd(OAc)_2_ as the catalyst, since it has stable catalytic working conditions even without the use of an inert atmosphere. The presence of the nucleophilic and electrophilic moieties in the same molecule, placed at the right distance and with the right orientation, is essential for an efficient DHA reaction but introduces the problem of the synthesis of the precursor. For this reason, the DHA intramolecular methodology is advantageous only in cases in which the precursor is obtained with simple reactions and in a few steps.

**Scheme 5:**
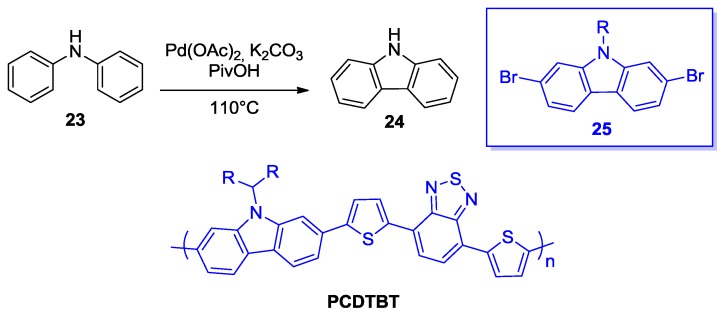
Intramolecular DHA reaction for the synthesis of carbazole monomers.

Fagnou et al. [36] reported an interesting carbazole synthesis using a mild and scalable intramolecular oxidative DHA reaction shown in Scheme 5. Carbazole monomers **25** have received much interest in the optoelectronic field due to highest occupied molecular orbital (HOMO) energy levels very close to the ideal for OPV applications. Carbazole scaffold **24** was obtained in 95% yield and without formation of byproducts, starting from diphenylamine **23** in presence of Pd(OAc)_2_ as the catalyst (3 mol %), K_2_CO_3_ as base (10 mol %) and PivOH as co-solvent to form **24** in air (O_2_ acts as the oxidant). The synthetic route developed provides the use of precursor **23**, scalable high efficiency, mild reaction conditions, and it avoids flash-chromatography purification.

**Scheme 6:**
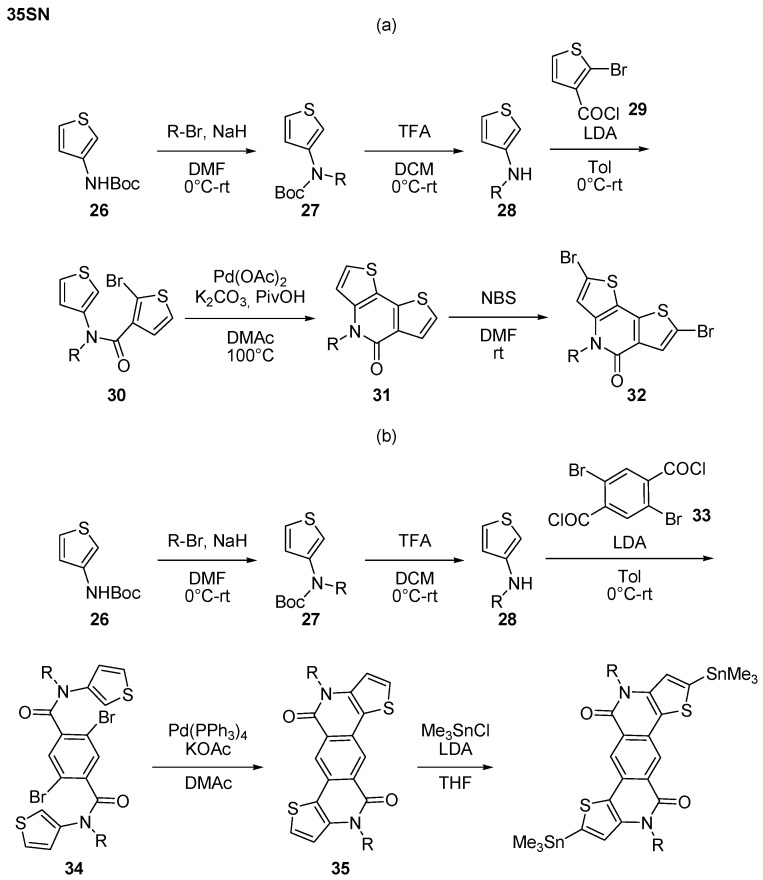
Synthesis of 2-pyridone dithiophene (PDT) and thieno pyrido thieno isoquinoline dione (TPTI) monomers. TFA: trifluoroacetic acid. (**a**) intramolecular DHA reaction for PDT monomer; (**b**) intramolecular DHA reaction for TPTI monomer.

Yu et al. [37] reported an interesting synthesis of 2-pyridone dithiophene (PDT) monomers using Pd(OAc)_2_-catalyzed intramolecular DHA reaction as the key step (Scheme 6a). The authors achieved the PDT synthesis in five steps via alkylation of Boc-protected 3-aminothiophene **26** (89% yield) followed by deprotection of the resulting alkylated amine **27** with trifluoroacetic acid (TFA). The deprotected amine **28** was directly reacted with thiophene **29** to afford precyclized intermediate **30** in 58% yield after three steps.

This compound was finally cyclized through an intramolecular DHA, that makes use of Pd(OAc)_2_ (10 mol %) as a catalyst and pivalic acid (30 mol %) as an additive, to give the PDT framework in excellent yields (90%, compound **31**). It was then brominated, using NBS, to obtain monomer **32**.

The same authors reported the synthesis of thieno pyrido thieno isoquinoline dione (TPTI) framework using a similar synthetic route (Scheme 6b) [38]. In this case the precyclized precursor **34** was obtained in a 52% yield by acylation reaction between **28** and compound **33**, while the intramolecular DHA of compound **34** was performed with Pd(PPh_3_)_4_ as a catalyst (4 mol %), and KOAc as a base in DMAc under diluted conditions (2 mM) to give the compound **35** in excellent yields (87%), which was then efficiently stannylated to obtain the polymerizable monomer. An easy and efficient synthesis of precyclized intermediate is fundamental to obtaining scalable monomers via DHA reactions. The synthetic design of monomers proposed by Yu take account of this aspect, in fact the synthesis of compounds **27** (81% yield), **28**, **30** (65% yield) and **34** (58% yield) make use of simple, cheap, scalable reactions.

**Scheme 7:**
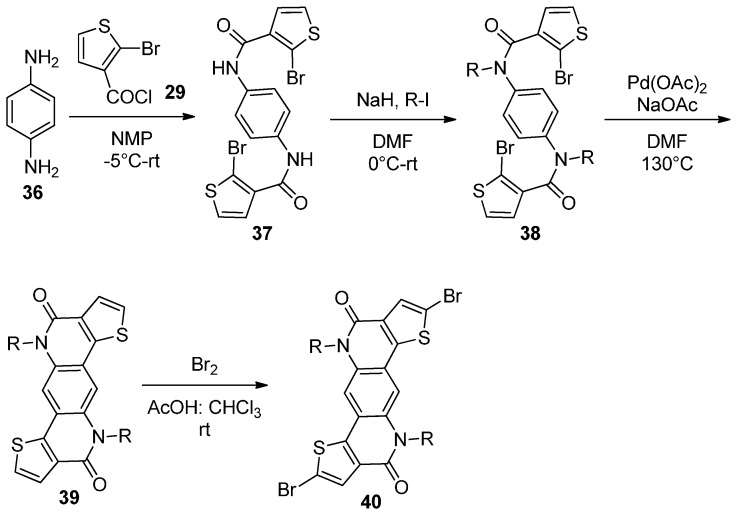
Synthesis of thiophene-phenylene-thiophene fused bilactam (TPTBL) monomer.

An easier synthetic design was proposed by Kim et al. [39] in the synthetic route shown in Scheme 7 for the synthesis of the novel class of acceptor monomers thiophene-phenylene-thiophene fused bilactam (TPTBL) **40**.

Starting from *p*-phenylenediamine **36** with 2-bromothiophene acid chloride **29** in NMP at temperature of −5 °C, bisamide **37** was obtained in 87% yield. The *N*-alkylation of bisamide **37** (70% yield) in the presence of NaH and alkyl iodide in DMF, followed by ring-closing Pd(OAc)_2_-catalyzed DHA reaction provides the desired thiophene-phenylene-thiophene fused bislactams TPTBL **39** in good yields (69%). Bromination reaction lead quantitatively to the TPTBL-polymerizable monomer **40**. The author incorporated intramolecular DHA reactions in a synthetic rational design that makes use of cheap reagents, reactions achieved in good or high yields, and a minimal number of synthetic steps.

**Scheme 8:**
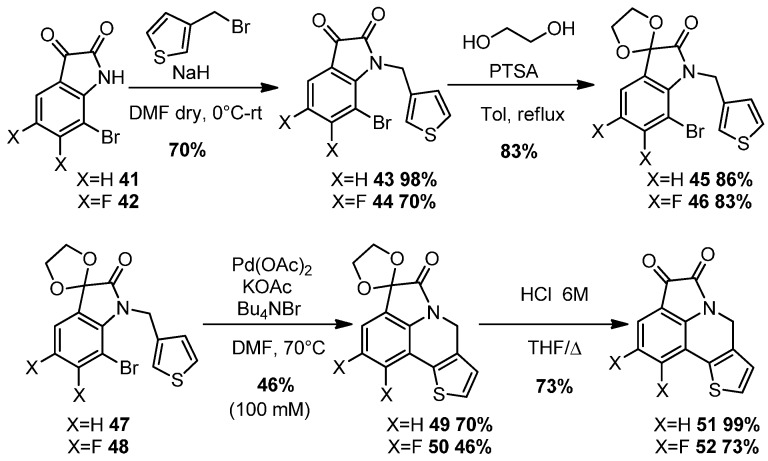
Synthesis of isatin-based dyes with push-pull architecture. PTSA: *p*-toluenesulfonic acid.

We have reported [40] the synthesis of conjugated π-extended dyes having push-pull architecture in which isatin, a naturally-occurring dye, is fused with a thiophene ring in four steps. The modification of natural pigments and dyes for the construction of π-conjugated compounds for electronic applications is increasingly popular, since natural dyes are often inexpensive and with optimal optical properties [41]. The synthesis of dyes **51** and **52** illustrated in Scheme 8, as in previous cases, adopts an intramolecular direct hetero-arylation (DHA) cyclization as the key step, in order to ensure potential for industrial scalability. NMR and X-ray studies demonstrate the crosstalk occurring between the fused, coplanar and conjugated moieties, making these novel dyes with a donor-acceptor character.

*N*-alkylation of bromoisatin derivatives **41** and **42** with 3-bromothiophene, conducted with NaH in DMF, followed by protection of C3 keto-carbonyl group with ethylene glycol in toluene on **43** and **44**, has led to *spiro*-dioxolane precyclized compounds **45** and **46** in high yields. Ring closing DHA reaction was carried out in the presence of Pd(OAc)_2_ as catalyst, an excess of potassium acetate as a base and in moderate dilution conditions to give *spiro*-dioxolane cyclized compounds **49** and **50** which were deprotected in quantitative yields in acidic conditions to obtain final dyes **51** and **52**.

**Scheme 9:**
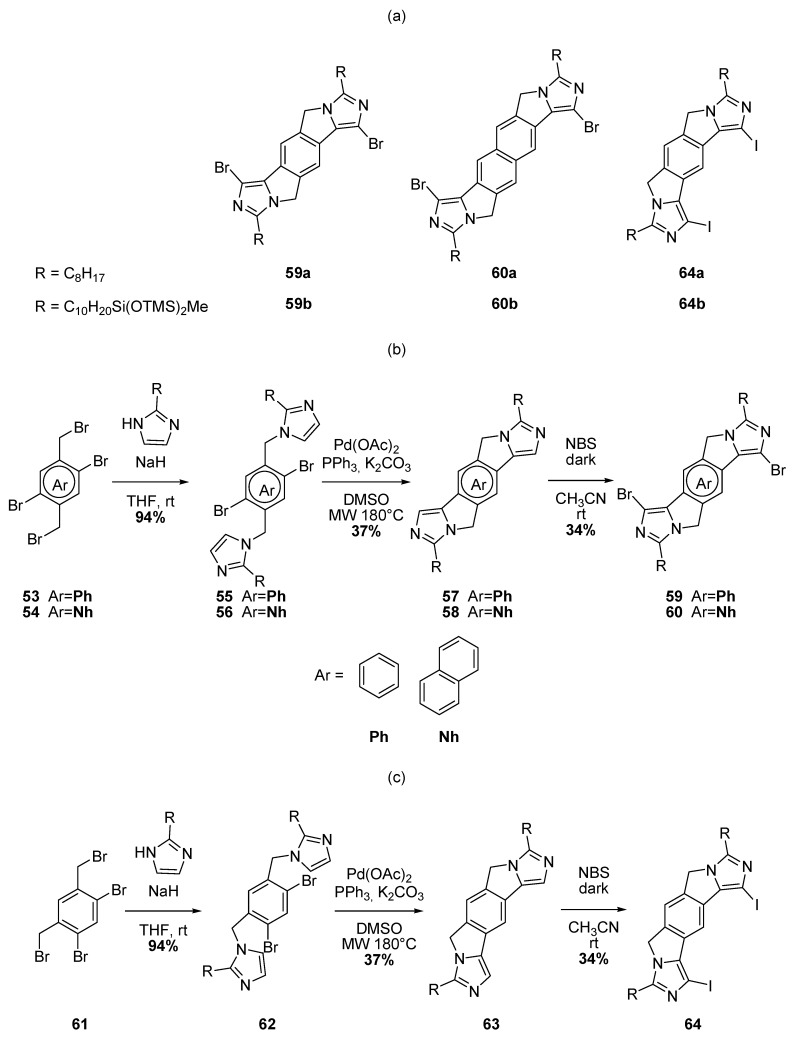
Synthesis of imidazole-based monomers. (**a**) π-conjugated fused imidazole monomers by microwave-assisted intramolecular DHA (**b**) intramolecular DHA for the synthesis of monomers **59** and **60** (**c**) intramolecular DHA for the synthesis of bent monomer **64**. NBS: *N*-bromosuccinimide.

A series of fully π-conjugated fused imidazole monomers bearing octyl chain or siloxane-terminated decyl chains as the solubilizing groups at the 2-position of the imidazole core were synthesized very recently by Masu et al. [42] utilizing the microwave-assisted intramolecular DHA (Scheme 9a). Few π-conjugated polymers composed of imidazole units in the main chain have been reported in literature to date [43,44,45]. The conjugated imidazole-based backbone proposed from Masu is particularly interesting because both the reactivity and basicity of imidazole unit are utilized to control the electronic and ordering structures of the polymers. In fact, the reactivity of the C5-H imidazole bond to give regioselective DHA with aryl halides or pseudohalides was used for construction of fully π-conjugated monomers **59a**–**b**, **60a**–**b** and **64a**–**b**; while the intrinsic basicity of the nitrogen atoms was used for post-protonation of the polymers, changing the pH of the solution, to modify the electronic, optical and ordering structure properties of the polymers.

Monomers **59a**–**b** and **60a**–**b** were synthesized according to synthetic route of four steps illustrated in Scheme 9b. *N*-alkylation of 2-alkyl-1*H*-imidazole with 1,4-dibromo-2,5-bis(bromomethyl)benzene **53** and 2,6-dibromo-3,7-bis(bromomethyl)naphthalene **54** with NaH in dry tetrahydrofuran (THF) at room temperature occurred smoothly without the need for chromatographic purifications, and pure precyclized compounds **55** and **56** were obtained in excellent yields. Intramolecular DHA reaction was carried out in the presence of Pd(OAc)_2_ and PPh_3_ as the catalyst system, an excess of potassium carbonate as base and DMSO at a temperature of 180 °C in MW-assisted conditions for 1.5 h to give imidazole fused compounds **57** and **58** in moderate yields. They were then brominated with NBS in CH_3_CN to obtain final monomers **59** and **60**. Bent monomer **64** was obtained following an analogous synthetic route, illustrated in Scheme 9c, starting from 1,5-dibromo-2,4-bis(bromomethyl)benzene **61**.

## 3. Conclusions

We have highlighted examples of the application of DHA methodologies as the key steps in the synthesis of π-extended monomers, suitable for polymerization to yield macromolecular scaffolds to be used in the field of OPV BHJ devices. When applicable, the DHA synthetic methodology bears substantial advantages over traditional methodologies, even if the yields are marginally reduced, owing to the atom economy concept lying behind it. The presented examples augur well for the full development of the field of OPV BHJ, for which the concepts of synthetic accessibility and scalability, in the context of bringing the technology to the market, will be more and more in demand.

## Abbreviations

BT	2,1,3-Benzothiadiazole
BTI	dithieno[3,2-*c*:2′,3′-*e*]azepine-4,6-dione
CPDT	4H-cyclopenta[1,2-*b*:5,4-*b*′]dithiophene
Cz	Carbazole
DBrBT	4,7-dibromo-2,1.3-benzothiadiazole
DEDOTBT	4,7-bis(3,4-ethylenedioxythiophene-2-yl)-2,1,3-benzothiadiazole
DHTBT	4,7-bis(4-hexylthiophen-2-yl)-2,1,3-benzothiadiazole
DMAc	*N*,*N*-dimethylacetamide
DMF	*N*,*N*-dimethylformamide
DTBDT	Dithienobenzodithiophene
DTBT	4,7-di(thiophen-2-yl)-2,1,3-benzothiadiazole
EDOT	3,4-ethylenedioxythiophene
F	Fluorene
HOMO	Highest occupied molecular orbital
HT	3-hexylthiophene
JHONPHOS	(2-Biphenyl)di-tert-butylphosphine
LUMO	Lowest unoccupied molecular orbital
NDT	Naphto[2,1-*b*:3,4-*b*′]dithiophene
NMP	N-methyl-2-pyrrolidone
ODMB	*o*-dimethylbenzene
P3HT	Poly(3-hexylthiophene)
PC_61_BM	[6,6]-phenyl-C61-butyric acid methyl esther
PC_71_BM	phenyl-[C71]-butyric acid methyl esther
PCE	Power conversion efficiency
PDT	2-pyridone dithiophene
PivOH	2,2-dimethylpropionic acid (Pivalic acid)
PSC	Polymer Solar Cell
T	Thiophene
TDPT	Thieno[2′,3′,:5,6]pyrido[3,4-*g*]thieno[2,2-*c*]isoquinoline-5,11-dione
TFA	Trifluoroacetic acid
TPTBL	Thiophene-phenylene-thiophene fused bilactam
TTe	Thieno[3,4-*b*]thiophene
Tz	Thiazole

## References

[1] Chiang C.K., Fincher C.R., Park Y.W., Heeger A.J., Shirakawa H., Louis E.J., Gau S.C., MacDiarmid A.G. (1977). Electrical Conductivity in Doped Polyacetylene. Phys. Rev. Lett..

[2] Shirota Y. (2000). Organic materials for electronic and optoelectronic devices. J. Mater. Chem..

[3] Wang C., Dong H., Hu W., Liu Y., Zhu D. (2012). Semiconducting π-Conjugated Systems in field-Effect Transistors: A Material Odyssey of Organic Electronics. Chem. Rev..

[4] Sasabe H., Kido J. (2013). Recent Progress in Phosphorescent Organic Light-Emitting Devices. Eur. J. Org. Chem..

[5] Lu L., Zheng T., Wu Q., Schneider A.M., Zhao D., Yu L. (2015). Recent Advances in Bulk Heterojunction Polymer Solar Cells. Chem. Rev..

[6] Clark J., Lanzani G. (2010). Organic photonics for communications. Nat. Photonics.

[7] Brabec C.J. (2004). Organic photovoltaics: Technology and market. Sol. Energy Mater. Sol. Cells.

[8] Liu Y., Zhao J., Li Z., Mu C., Ma W., Hu H., Jiang K., Lin H., Ade H., Yan H. (2014). Aggregation and Morphology Control Enables Multiple cases of High-Efficiency Polymer Solar Cells. Nat. Commun..

[9] Li G., Zhu R., Yang Y. (2012). Polymer Solar Cells. Nat. Photonics.

[10] Po R., Bernardi A., Calabrese A., Carbonera C., Corso G., Pellegrino A. (2014). From lab to fab: How must the polymer solar cell materials design change?—An industrial perspective. Energy Environ. Sci..

[11] Jørgensen M., Carlé J.E., Søndergaard R.R., Lauritzen M., Dagnæs-Hansen N.A., Byskov S.L., Andersen T.R., Larsen-Olsen T.T., Böttiger A.P.L., Andreasen B. (2013). The state of organic solar cells—A meta analysis. Sol. Energy Mater. Sol. Cells.

[12] Maluenda I., Navarro O. (2015). Recent Developments in the Suzuki-Miyaura Reaction: 2010–2014. Molecules.

[13] Cordovilla C., Bartolome C., Martínez-Ilarduya J.M., Espinet P. (2015). The Stille Reaction, 38 Years Later. ACS Catal..

[14] Morin P.-O., Bura T., Leclerc M. (2016). Realizing the full potential of conjugated polymers: Innovation in polymer synthesis. Mater. Horiz..

[15] Po R., Bianchi G., Carbonera C., Pellegrino A. (2015). “All That Glisters Is Not Gold”: An Analysis of the Synthetic Complexity of Efficient Polymer Donors for Polymer Solar Cells. Macromolecules.

[16] Okamoto K., Zhang J., Housekeeper J.B., Marder S.R., Luscombe C.K. (2013). C–H Arylation Reaction: Atom Efficient and Greener Syntheses of π-Conjugated Small Molecules and Macromolecules for Organic Electronic Materials. Macromolecules.

[17] Schipper D.J., Fagnou K. (2011). Direct Arylation as a Synthetic Tool for the Synthesis of Thiophene-Based Organic Electronic Materials. Chem. Mater..

[18] Kudrjasova J., Kesters J., Verstappen P., Brebels J., Vangerven T., Cardinaletti I., Drijkoningen J., Penxten H., Manca J., Lutsen L. (2016). A direct arylation approach towards efficient small molecule organic solar cells. J. Mater. Chem. A.

[19] Lin P.-H., Lu T.-J., Cai D.-J., Lee K.-M., Liu C.-Y. (2015). Connecting Direct C–H Arylation Reactions with Dye-Sensitized Solar Cells: A Shortcut to D–A–π–A Organic Dyes. ChemSusChem.

[20] Hou J., Park M.-H., Zhang S., Yao Y., Chen L.-M., Li J.-H., Yang Y. (2008). Bandgap and Molecular Energy Level Control of Conjugated Polymer Photovoltaic Materials Based on Benzo[1,2-*b*:4,5-*b*′]dithiophene. Macromolecules.

[21] Livi F., Zawacka N.K., Angmo D., Joergensen M., Krebs F.C., Bundgaard E. (2015). Influence of Side Chain Position on the Electrical Properties of Organic Solar Cells Based on Dithienylbenzothiadiazole-alt-phenylene Conjugated Polymers. Macromolecules.

[22] Wang Z., Liu Q., Chen T., Wang Y., Yuan J., Zheng C., Chen R., Huang W. (2015). Molecular rearrangement at charged states: Intrinsic effects upon photo and electroluminescence. Dyes Pigment..

[23] Melkonyan F.S., Zhao W., Dress M., Eastham N.D., Leonardi M.J., Butler M.R., Chen Z., Yu X., Chang R.P.H., Ratmer M.A. (2016). Bithiophenesulfonamide Building Block for π-Conjugated Donor–Acceptor Semiconductors. J. Org. Chem. Soc..

[24] Tovar J.D., Rose A., Swager T.M. (2002). Functionalizable Polycyclic Aromatics through Oxidative Cyclization of Pendant Thiophenes. J. Am. Chem. Soc..

[25] Alberico D., Scot M.E., Lautens M. (2007). Aryl–Aryl Bond Formation by Transition-Metal-Catalyzed Direct Arylation. Chem. Rev..

[26] Mercier L.G., Leclerc M. (2013). Direct(Hetero)Arylation: A New Tool for Polymer Chemists. Acc. Chem. Res..

[27] Chang S.-W., Waters H., Kettle J., Horie M. (2012). Cyclopentadithiophene–benzothiadiazole oligomers: Synthesis via direct arylation, X-ray crystallography, optical properties, solution casted field-effect transistor and photovoltaic characteristics. Org. Electron..

[28] Chang S.-W., Waters H., Kettle J., Kuo Z.-R., Li C.-H., Yu C.-Y., Horie M. (2012). Pd-catalysed direct arylation polymerisation for synthesis of low-bandgap conjugated polymers and photovoltaic performance. Macromol. Rapid Commun..

[29] Wang M., Ford M., Phan H., Coughlin J., Nguyena T.-Q., Bazan G.C. (2016). Fluorine substitution influence on benzo[2,1,3]thiadiazole based polymers for field-effect transistor applications. Chem. Commun..

[30] Sharma B., Sarothia Y., Singh R., Kan Z., Keivanidis P.E., Jacob J. (2016). Synthesis and Characterization of Light-Absorbing Cyclopentadithiophene-Based Donor–Acceptor Copolymers. Polym. Int..

[31] Wang X., Wang K., Wang M. (2015). Synthesis of Conjugated Polymers via an Exclusive Direct-Arylation Coupling Reaction: A Facile and Straightforward Way to Synthesize Thiophene-Flanked Benzothiadiazole Derivatives and their Copolymers. Polym. Chem..

[32] Hou Q., Xu Y.S., Yang W., Yuan M., Peng J.B., Cao Y. (2002). Novel Red-Emitting Fluorene-Based Copolymers. J. Mater. Chem..

[33] Liu B., Najari A., Pan C.Y., Leclerc M., Xiao D.Q., Zou Y.P. (2010). New Low Bandgap Dithienylbenzothiadiazole Vinylene Based Copolymers: Synthesis and Photovoltaic Properties. Macromol. Rapid Commun..

[34] Chàvez P., Ngov C., Frémont P., Lévêque P., Leclerc N. (2014). Synthesis by Direct Arylation of Thiazole–Derivatives: Regioisomer Configurations–Optical Properties Relationship Investigation. J. Org. Chem..

[35] Guo Q., Jiang R., Wu D., You J. (2016). Rapid Access to 2,2′-Bithiazole-Based Copolymers via Sequential Palladium-Catalyzed C–H/C–X and C–H/C–H Coupling Reactions. Macromol. Rapid Commun..

[36] Liégault B., Lee D., Huestis M.P., Stuart D.R., Fagnou K. (2008). Intramolecular Pd(II)-Catalyzed Oxidative Biaryl Synthesis Under Air: Reaction Development and Scope. J. Org. Chem..

[37] Schneider A.M., Lu L., Manley E.F., Zheng T., Sharapov V., Xu T., Marks T.J., Chen L.X., Yu L. (2015). Wide bandgap OPV polymers based on pyridinonedithiophene unit with efficiency >5%. Chem. Sci..

[38] Jung I.H., Lo W.-Y., Jang J., Chen W., Zhao D., Landry E.S., Lu L., Talapin D.V., Yu L. (2014). Synthesis and Search for Design Principles of New Electron Accepting Polymers for All-Polymer Solar Cells. Chem. Mater..

[39] Poduval M.K., Burrezo P.M., Cadaso J., López Navarrete J.T., Ortiz R.P., Kim T.-H. (2013). Novel Thiophene–Phenylene–Thiophene Fused Bislactam-Based Donor–Acceptor Type Conjugate Polymers: Synthesis by Direct Arylation and Properties. Macromolecules.

[40] Nitti A., Signorile M., Boiocchi M., Bianchi G., Po R., Pasini D. (2016). Conjugated Thiophene-Fused Isatin Dyes through Intramolecular Direct Arylation. J. Org. Chem..

[41] Gsänger M., Bialas D., Huang L., Stolte M., Würthner F. (2016). Organic Semiconductors based on Dyes and Color Pigments. Adv. Mater..

[42] Takagi K., Miwa T., Masu H. (2016). Synthesis and Optical Properties of π-Conjugated Polymers Containing Fused Imidazole Skeleton. Macromolecules.

[43] Tennyson A.G., Kamplain J.W., Bielawski C.W. (2009). Oxidation of Poly(enetetramine)s: A New Strategy for the Synthesis of Conjugated Polyelectrolytes. Chem. Commun..

[44] Tennyson A.G., Norris B., Bielawski C.W. (2010). Structurally Dynamic Conjugated Polymers. Macromolecules.

[45] Neilson B.M., Tennyson A.G., Bielawski C.W. (2012). Advances in Bis(N-heterocyclic carbene) Chemistry: New Classes of Structurally Dynamic Materials. J. Phys. Org. Chem..

